# Obinutuzumab as a viable therapeutic strategy in rituximab-refractory childhood frequently relapsing, steroid-dependent nephrotic syndrome that relapsed during B-cell depletion

**DOI:** 10.1007/s00467-024-06570-8

**Published:** 2024-10-28

**Authors:** Eugene Yu-hin Chan, Kyle Ying-kit Lin, Desmond Yat-hin Yap, Alison Lap-tak Ma

**Affiliations:** 1 Department of Paediatrics, Faculty of Medicine, The Chinese University of Hong Kong, Shatin, Hong Kong SAR; 2 Paediatric Nephrology Centre, Hong Kong Children’s Hospital, Kowloon, Hong Kong SAR; 3 Division of Nephrology, Department of Medicine, Queen Mary Hospital, The University of Hong Kong, Hong Kong, Hong Kong SAR

**Keywords:** Nephrotic syndrome, Rituximab, Obinutuzumab, Anti-CD20, Focal segmental glomerulosclerosis

## Abstract

A subgroup of children with frequently-relapsing, steroid-dependent nephrotic syndrome relapse during B-cell depletion after rituximab. A 15-year-old boy with focal segmental glomerulosclerosis became rituximab-refractory after 5 courses of treatments, with a relapse-free period shortened to 1 month. Circulating total and memory B-cells were undetectable at the time of relapse. A single infusion of obinutuzumab sustained relapse-free remission up to the last follow-up at 18 months. There was persistent hypogammaglobulinemia but no infection was observed. Obinutuzumab may be a viable option for attaining long-term remission with reasonable side effect profiles in patients who relapse during B-cell depletion after rituximab.

## Introduction

Rituximab is an important treatment for frequently relapsing, steroid-dependent nephrotic syndrome (FRSDNS). This indicates the role of B-cells in the pathogenesis of NS. Nonetheless, some children suffer from relapses despite successful B-cell depletion [1], which constitutes a major challenge in disease management. We report for the first time the use of second-generation anti-CD20 antibodies, obinutuzumab, as a viable strategy to sustain long-term remission in this scenario.

## Case presentation

Our patient was a 15-year-old boy who presented with steroid-sensitive nephrotic syndrome at 3 years of age. A kidney biopsy revealed focal segmental glomerulosclerosis (FSGS). He developed repeated relapses despite receiving long-term prednisolone and multiple steroid-sparing agents. These included levamisole, cyclophosphamide, mycophenolate mofetil (MMF), cyclosporine A and tacrolimus. He suffered from significant treatment-associated adverse effects such as Cushing syndrome, short stature and osteoporosis.

At 9 years old, he was referred to our care and received rituximab at 375 mg/m^2^ for two infusions, followed by maintenance MMF. He attained B-cell depletion and sustained remission for 7.6 months post-rituximab. Since then, he received multiple courses of rituximab for relapses and achieved remission for up to 13 months. At 13.5 years, he was given the sixth course of rituximab prophylactically, to optimise pubertal growth, with maintenance prednisolone and tacrolimus. The prednisolone dose was 5 mg per day, while tacrolimus was prescribed at 1.5 mg per day, with a 12-h trough level of 3.2 µg/L. However, this infusion was only able to sustain the remission for 4.6 months. Importantly, upon relapse, both total B-cells (CD19 + ; < 0.05%) and memory B-cells (CD19 + CD27 + ; < 0.05%) remained to be depleted. Total B-cells subsequently repopulated at 8 months post-rituximab. While awaiting obinutuzumab to be introduced into the hospital’s drug formulary, he received an additional course of rituximab with triple immunosuppression (prednisolone, MMF and tacrolimus). Again, despite successful depletion of total and memory B cells, he developed another relapse within 1 month.

**Fig. 1:**
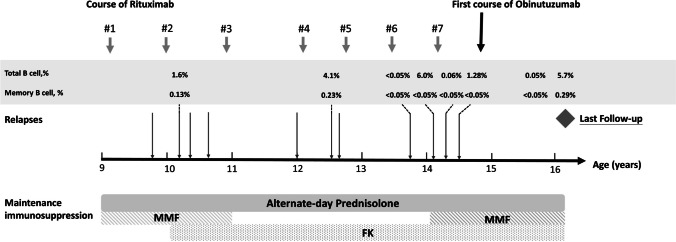
Clinical course between the first administration of rituximab therapy, first infusion of obinutuzumab and last follow-up. Relapse, urine protein: creatinine ratio > 2 mg/mg. MMF, mycophenolate mofetil; FK, tacrolimus

A single infusion of obinutuzumab (1000 mg/1.73 m^2^) was given 8 months after the previous course of rituximab. Pre-medications included intravenous methylprednisolone (100 mg/dose), intravenous chlorpheniramine (10 mg/dose) and oral paracetamol 500 mg. The infusion rate was slowly increased from 0.2 to a maximum of 2 mg/kg/hour. No infusion reaction was observed. Cotrimoxazole was given for 6 months as prophylaxis against Pneumocystis jirovecii. Prior to obinutuzumab infusion, total and memory B-cells were 4% and 0.13% of the total lymphocyte count, respectively. There was pre-existing hypogammaglobulinaemia at the time of obinutuzumab (IgG, 1.01 g/L, reference 5.95–13.10; IgM 0.30 g/L, reference 0.15–1.88; IgA 1.28 g/L, reference 0.47–2.49). B-cell depletion was documented 4 weeks later (total B-cell, 0.05%; memory B-cells, < 0.05%). Due to the refractory disease course, concurrent immunosuppressants prescribed long before the use of obinutuzumab were continued. The clinical course between the first administration of rituximab and last follow-up is shown in Fig. 1.

At 18 months post-obinutuzumab, he remained well in disease remission and prednisolone was tapered to 10 mg alternate days. Total B-cells and memory B-cells were 0.05% and < 0.05% at 12 months and started to repopulate (5.71% and 0.29%, respectively) upon the last follow-up at 18 months. Hypogammaglobulinaemia persisted over the observation period. While there was a slight improvement in IgG levels, the levels of IgM became undetectable from 3 months post-obinutuzumab and reconstituted at the last follow-up (IgG, 1.83 g/L; IgM, 0.46 g/L; IgA, 1.31 g/L). The patient, however, did not develop infection, neutropenia or other significant adverse events. Intravenous immunoglobulin replacement was not given. There was no EBV or CMV infection. Table 1 summarises the clinical and laboratory findings after obinutuzumab.

**Table 1:** Treatment response, B-cell immunophenotyping profiles and laboratory values following first course of obinutuzumab

First course of obinutuzumab at 14.8 years (1000 mg/1.73 m^2^)																	
Time from obinutuzumab	Remission status	Maintenance immunosuppression			B cells (CD19 +)/total lymphocytes	Memory B cells (CD19 + CD27 +)/total lymphocytes	WBC (× 10^9^/L)	Lymphocyte (× 10^9^/L)	Neutrophil (× 10^9^/L)	Haemogloblin (g/dL)	Platelet (× 10^9^/L)	ALT	Creatinine (µmol/L)	Tacrolimus (ug/L)	IgA (g/L)	IgG (g/L)	IgM (g/L)
		Prednisolone, mg/day	MMF, mg/day	FK, mg/day													
At baseline	In remission	40	1000	2.5	4%	0.13%	26.2	2.49	22.7	14.1	446	< 7	48	5.0	1.28	1.01	0.3
1 month	No relapse	10	1000	2	0.05%	< 0.05%	9.86	0.97	8.75	13.9	418	22	55	4.9	0.97	1.54	0.1
3 months	No relapse	5	1000	2	0.09%	< 0.05%	14.1	3.03	9.41	14.1	554	< 7	55	5.0	0.75	2.05	< 0.06
6 months	No relapse	5	1000	2	0.07%	< 0.05%	12.09	3.37	7.2	13.8	612	11	53	4.6	0.63	1.99	< 0.66
9 months	No relapse	5	1000	2	< 0.05%	< 0.05%	10.91	2.46	6.7	13	509	11	53	3.4	0.64	1.88	< 0.06
12 months	No relapse	5	1000	2	0.05%	< 0.05%	8.05	3.31	3.09	13.9	486	14	54	4.2	0.55	1.83	< 0.06
15 months	No Relapse	5	1000	2	3.9%	0.05%	11.47	4.47	5.16	14.6	438	7	55	1.4	-	-	-
18 months	No relapse	5	1000	2	5.71%	0.29%	8.82	3.29	3.73	15.2	396	< 7	47	2.4	1.31	1.83	0.49

*FK* tacrolimus, *MMF* mycophenolate mofetil, *WBC* white blood cells, *ALT* alanine aminotransferase, *IgA* immunoglobulin A, *IgG* immunoglobulin G, *IgM* immunoglobulin M

## Discussion

We first describe the use of obinutuzumab to overcome the challenge of multidrug-dependent FRSDNS that relapses despite B-cell depletion after rituximab, sustained remission with reasonable side effect profiles.

Mounting evidence has shown the efficacy of rituximab in NS through restoring B- and T-cell immune homeostasis directly and indirectly [2]. Earlier reports demonstrated that NS relapses often occurred after total B-cell reconstitution [2]. Nonetheless, there is no substantial temporal relationship between total B-cells and relapse. Recent studies reveal that memory B-cells, a subset of the B-cell lineage, is closely associated with relapse [2]. Our patient experienced NS relapses, despite undetectable circulating levels of total and memory B-cells (< 0.05%), following two distinct courses of rituximab. This observation had been described by Sato et al., where 7% patients relapsed during B-cell depletion post-rituximab [1]. This phenomenon is thought to indicate that mechanistic pathways other than B-cell immunity are implicated in a subset of children with NS. Importantly, there is currently no effective treatment for this therapeutic dilemma.

Obinutuzumab is a type II anti-CD20 monoclonal antibody that is associated with a more profound depletion of B-cells and their subsets in both blood and tissue. In our patient, the last two courses of rituximab were ineffective, and the relapse-free periods were short—between 1 and 5 months—even in the presence of triple maintenance immunosuppression acting on the T-cell immunity. We speculate that in our patient, rituximab did not deplete memory B-cells residing within tissue and secondary lymphoid organs, such as lymph nodes and spleen [3], which continued to produce pathogenic circulating factors. The use of obinutuzumab enables more effective suppression of circulating factors, thereby achieving sustained disease remission. While measurements of anti-nephrin antibodies and anti-rituximab antibodies were unavailable in our locality, assessment of these biomarkers would have helped to better understand the case. One potential confounder was the use of concomitant triple immunosuppression, although the patient also received triple immunosuppression during the last course of rituximab, with trough tacrolimus levels comparable to that during obinutuzumab therapy. Following obinutuzumab, the family opted to continue triple immunosuppressants since previous relapses were associated with the development of acute kidney injuries and our patient had significant corticosteroid toxicities. Recently, Dossier et al. reported their experience in treating FRSDNS with obinutuzumab [4]. Of the 41 children with rituximab-refractory FRSDNS receiving obinutuzumab, 92% and 68% subjects were in sustained remission at 12 and 24 months. The study, however, did not include children who relapsed during circulating B-cell depletion.

Safety profiles appear to be acceptable in our patient. Apart from prolonged hypogammaglobulinemia of very low levels, there was no clinical infection and other reported adverse events. Hypogammaglobulinaemia occurs in 14–58% children treated with rituximab for NS [5]. In concordance with the aforementioned report on obinutuzumab [4], our patient developed significantly low levels of IgM, while IgG showed a gradual increment over time. Of note, our patient also had low levels of IgG prior to obinutuzumab which might contribute to the development and perpetuation of hypogammaglobulinemia [5]. Reduction of IgM levels has also been reported in up to 46% of patients treated with rituximab for various childhood conditions [6]. Importantly, low IgM levels may potentially contribute to infections [6]. Intravenous immunoglobulin replacement was not prescribed in our case, since only 1% of hypogammaglobulinaemia episodes develop infection [5], and current recommendations do not support substitution for asymptomatic hypogammaglobulinaemia. Furthermore, intravenous immunoglobulin contains only a minimal amount of IgM, which limits its use for preventing infections. The use of a prophylactic antibiotic is recommended for 3–6 months following anti-CD20 therapy [2]. Co-trimoxazole was given for 6 months, according to the departmental protocol, in our patient to prevent Pneumocystis jirovecii infection. In view of the longer duration of B-cell depletion with obinutuzumab, it is justifiable to extend its use until B-cell repopulation. While obinutuzumab appeared to be a feasible therapeutic option in this specific clinical setting, the decision to prescribe obinutuzumab should be excised with caution due to potential risk of severe infection, particularly in the presence of pre-existing hypogammaglobulinemia and concomitant immunosuppression.

In conclusion, obinutuzumab could be a viable option to achieve durable remission, more profound/complete B-cell depletion and corticosteroid-sparing effects in patients with rituximab-refractory NS who relapse despite circulating B-cell depletion. Future trials are required to establish the role of obinutuzumab in this difficult-to-treat disease.

## Summary

### What is new?

- Rituximab-refractory nephrotic syndrome that relapses during B-cell depletion constitutes a major therapeutic challenge. The use of obinutuzumab is associated with sustained disease remission, prolonged B-cell depletion and room for corticosteroid tapering.

## Data Availability

Researchers interested in using more detailed patient-level data could obtain them upon request to the corresponding authors.
